# U.S. Emergency Department Visits for Dizziness and Vertigo From 2016 to 2022

**DOI:** 10.1111/acem.70214

**Published:** 2026-01-13

**Authors:** Milina M. Miulli, Howard S. Kim

**Affiliations:** ^1^ Department of Emergency Medicine Northwestern University Feinberg School of Medicine Chicago Illinois USA; ^2^ Center for Dissemination & Implementation Science Northwestern University Feinberg School of Medicine Chicago Illinois USA

Dizziness is a common symptom that affects 37 million Americans annually and frequently results in visits to the emergency department (ED) [1]. For clinicians, dizziness can be a difficult patient complaint to diagnose because of its numerous potential causes, including vestibular and non‐vestibular medical diagnoses. Thus, ED visits for dizziness commonly result in unnecessary diagnostic testing, which contributes to increased healthcare costs with limited added benefit [2]. National estimates of ED visits for dizziness have not been updated since 2008 [3]; this study therefore aims to describe contemporary trends in usual emergency care for dizziness using the most recent National Hospital Ambulatory Medical Care Survey (NHAMCS) data. NHAMCS is a nationally representative, annual survey of U.S. ED visits in which sampling weights adjust for selection probability and nonresponse, allowing estimation of national visit volumes and characteristics.

We included ED visits from 2016 to 2022 if one of the first 3 “reason for visit” (RFV) codes was for dizziness (1225.0 Vertigo—dizziness), which includes: falling sensation, dizziness, lightheadedness, loss of sense of equilibrium or balance, and room spinning. We selected a 2016 study start based on the transition to ICD‐10 in this year; 2022 was the last year of NHAMCS data collection. Since our objective was to describe usual emergency care for dizziness, we chose care variables that would be useful for operational benchmarking and identifying targets for future research and quality improvement: rates of head computed tomography (CT) and magnetic resonance imaging (MRI) use, administered and prescribed medications, and ED length of stay. For medications, we focused on administration and prescribing for the following medications previously described in dizziness literature: antihistamines, antiemetics, benzodiazepines, and glucocorticoids [4, 5]. The full list of variables is presented in Table 1, with corresponding point estimates obtained via the *svyset* package in StataMP 18.0. Detailed cohort selection criteria and care variable definitions are described in eMethods. Unweighted counts represent ED visits sampled directly by the NHAMCS survey, while weighted estimates approximate national visit volumes based on survey design.

**TABLE 1:** Unadjusted demographic and clinical characteristics of ED visits dizziness, 2016–2022.

	2016	2017	2018	2019	2020	2021	2022	Total
Unweighted count all ED visits	19,467	16,709	20,291	19,481	14,860	16,207	16,025	123,040
Weighted count all ED visits, in thousands	145,591	138,977	129,974	150,650	131,297	139,781	155,398	991,668
Unweighted count dizziness visits	692	525	673	689	497	549	533	4158
Weighted count dizziness visits, in thousands	5271	4294	4447	5496	4338	4823	5004	33,673
Proportion of all weighted ED visits due to dizziness	3.6%	3.1%	3.4%	3.6%	3.3%	3.5%	3.2%	3.4%
Age; mean	49.5	50.3	49.3	50.0	52.5	52.1	50.81	50.6
Age categories, %								
≤ 15 years	2.7%	2.3%	5.6%	4.2%	3.7%	4.0%	5.6%	4.0%
15–24 years	14.2%	11.2%	12.0%	11.1%	7.6%	8.9%	11.0%	10.9%
25–44 years	26.4%	28.1%	25.3%	27.0%	23.6%	26.2%	24.2%	25.9%
45–64 years	27.5%	29.6%	28.8%	26.6%	32.9%	28.5%	26.2%	28.4%
65–74 years	13.9%	12.3%	13.3%	16.4%	16.1%	12.2%	16.9%	14.5%
≥ 75 years	15.4%	16.6%	15.1%	14.7%	16.2%	20.2%	15.9%	16.3%
Sex, %								
Female	60.9%	56.1%	62.7%	58.9%	61.7%	62.5%	57.0%	60.0%
Male	39.1%	44.0%	37.3%	41.1%	38.3%	37.5%	43.0%	40.1%
Race/ethnicity, %								
Non‐Hispanic White	63.4%	55.3%	54.1%	56.5%	60.3%	61.5%	59.0%	58.7%
Non‐Hispanic Black	18.4%	22.7%	26.3%	22.8%	21.3%	20.0%	19.0%	21.4%
Hispanic	14.4%	17.8%	14.4%	16.8%	14.4%	15.1%	16.6%	15.6%
Non‐Hispanic Other	3.8%	4.3%	5.2%	3.9%	4.4%	3.5%	5.4%	4.3%
Region, %								
Northeast	15.4%	16.7%	17.3%	16.2%	21.4%	17.8%	18.3%	17.5%
Midwest	26.4%	28.7%	20.9%	20.0%	16.8%	21.1%	23.8%	22.5%
South	32.8%	37.0%	40.9%	40.2%	36.0%	38.8%	29.6%	36.4%
West	25.5%	17.6%	20.9%	23.7%	25.9%	22.3%	28.2%	23.6%
Primary Insurance^a^, %								
Private	25.4%	23.1%	26.8%	21.9%	22.6%	26.7%	25.7%	24.6%
Medicare	29.5%	31.4%	29.1%	29.9%	30.9%	32.3%	29.4%	30.3%
Medicaid	25.8%	26.7%	23.8%	22.8%	23.0%	23.4%	27.2%	24.6%
Worker's compensation	1.2%	0.2%	0.3%	0.01%	0.7%	0.0%	0.1%	0.4%
Self‐pay	4.9%	6.9%	5.9%	5.7%	7.4%	5.1%	4.2%	5.6%
No charge	0.5%	1.3%	0.2%	0.0%	0.6%	0.1%	0.2%	0.4%
Other/unknown	11.8%	9.6%	13.0%	15.1%	14.6%	9.2%	13.0%	12.4%
# of chronic conditions^b^, mean	1.4	1.6	1.5	1.4	1.7	1.8	1.6	1.6
Episode of care, %								
Initial	88.8%	90.7%	86.3%	89.1%	85.9%	83.6%	81.7%	86.6%
Follow‐up	3.6%	4.0%	6.1%	3.5%	6.4%	3.8%	5.2%	4.6%
Unknown or blank	7.2%	5.0%	6.3%	4.4%	5.1%	10.6%	11.8%	7.3%
Arrival by ambulance	21.7%	28.1%	18.8%	19.6%	27.9%	25.2%	21.1%	23.0%
Triage immediacy								
ESI 1: immediate	0.2%	0.0%	0.3%	0.6%	0.2%	2.7%	0.7%	0.7%
ESI 2: emergent	10.6%	19.6%	12.8%	9.1%	19.5%	17.6%	14.6%	14.5%
ESI 3: urgent	44.5%	43.2%	46.9%	48.9%	42.4%	46.0%	47.0%	45.7%
ESI 4: semi‐urgent	12.7%	8.6%	8.3%	6.3%	6.1%	4.9%	7.8%	7.9%
ESI 5: non‐urgent	1.8%	1.0%	1.1%	1.5%	0.3%	0.9%	0.0%	1.0%
Unknown/blank/no triage	30.2%	27.5%	30.7%	33.6%	31.6%	28.0%	29.8%	30.3%
Wait time in minutes^c^, mean	43.4	44.3	40.8	45.3	38.3	35.4	34.0	40.2
Seen by resident	7.1%	10.6%	9.5%	9.2%	11.3%	10.3%	10.8%	9.8%
Seen by advanced practice provider	23.7%	18.1%	21.2%	24.9%	27.5%	26.6%	25.5%	24.0%
Medications administered								
Antiemetic	31.8%	31.2%	29.6%	28.7%	29.7%	28.1%	28.8%	29.7%
Antihistamine	6.1%	5.7%	6.2%	3.3%	4.8%	5.0%	5.5%	5.2%
Benzodiazepine	6.4%	4.8%	5.8%	6.1%	6.4%	3.4%	4.1%	5.3%
Glucocorticoid	6.4%	4.5%	6.2%	3.3%	2.7%	3.0%	2.7%	4.1%
Medications prescribed								
Antiemetic	21.3%	15.8%	16.0%	15.8%	12.9%	14.2%	16.9%	16.3%
Antihistamine	4.0%	2.1%	2.2%	2.5%	0.6%	3.8%	2.1%	2.5%
Benzodiazepine	2.4%	1.9%	2.3%	2.1%	1.3%	0.4%	3.1%	2.0%
Glucocorticoid	2.9%	3.6%	3.6%	2.0%	1.0%	2.4%	1.5%	2.4%
Medications administered or prescribed								
Antiemetic	39.1%	35.9%	34.5%	33.5%	34.0%	33.2%	35.2%	35.1%
Antihistamine	9.5%	7.2%	7.4%	4.6%	5.1%	7.1%	8.7%	7.1%
Benzodiazepine	7.5%	5.8%	7.3%	6.7%	6.9%	4.0%	5.1%	6.2%
Glucocorticoid	4.2%	1.9%	3.2%	2.1%	1.8%	1.8%	1.2%	2.3%
Diagnostic imaging obtained								
Computed tomography (CT)	34.1%	27.3%	25.8%	28.5%	24.9%	30.9%	26.1%	28.4%
Magnetic resonance imaging	3.9%	3.3%	2.1%	3.3%	4.6%	4.7%	2.9%	3.6%
Length of visit in minutes^d^, mean	N/A	N/A	249.7	247.8	325.4	376.2	312.4	301.2

*Note:* All variables are weighted unless otherwise noted as unweighted.

^a^ Primary Insurance: In NHAMCS, dual eligible enrollees are classified as having primary Medicare insurance.

^b^ Chronic conditions include: alcohol abuse or dependence, Alzheimer's, asthma, cancer, cerebrovascular disease, chronic kidney disease, chronic obstructive pulmonary disease, congestive heart failure, coronary artery disease, depression, diabetes, end‐stage renal disease, history of pulmonary embolism or deep vein thrombosis, HIV, hyperlipidemia, hypertension, obesity, obstructive sleep apnea, osteoporosis, and substance abuse or dependence.

^c^ Wait Time is only available for *n* = 30,561,935 weighted visits (90.8%), *n* = 3699 unweighted visits (89.0%).

^d^ Length of Visit is not available in NHAMCS in 2016 and 2017 due to “data quality issues” in these years. Length of Visit is therefore only available for *n* = 24,107,862 weighted visits (71.6%), *n* = 2941 unweighted visits (70.7%).

To assess variation by age, an important factor influencing imaging and medication decisions, we constructed survey weighted multivariable logistic regression models to obtain predicted probabilities for the three outcomes of head CT receipt, antihistamine administration or prescription, and benzodiazepine administration or prescription—adjusting for year, repeat ED visit, number of co‐morbidities, and evaluation by either an advanced practice provider or resident physician. We selected these covariates based on prior literature [6] and NHAMCS visit characteristics with strong face validity. We selected these three outcomes for the adjusted analysis given contemporary interest in diagnostic imaging appropriateness and medications associated with falls [4]. Odds ratios from the logistic regression models were transformed to average predicted probabilities using Stata's postestimation margin commands. Because marginal estimates require stratification on an independent variable, we selected age categories as we felt this was the most intuitive way to contextualize rates of diagnostic imaging and medication use.

Between 2016 and 2022 (Table 1), there were 4158 unweighted ED visits for dizziness, representing a weighted estimate of 33.7 million ED visits for dizziness (4.8 million annual visits) or 3.4% of all ED visits. The mean age of ED dizziness visits was 50.6 years; 60.0% were female, and 58.7% were non‐Hispanic White; the most common insurance was Medicare (30.3%), followed by commercial insurance and Medicaid (both 24.6%). The mean wait time and length of stay were 40.2 min and 301.2 min, respectively; 23.0% of visits arrived by ambulance. CT and MRI utilization rates were 28.4% and 3.6%, respectively. Antiemetics were the most common medication administered or prescribed (35.1%), followed by antihistamines (7.1%) and benzodiazepines (6.2%). Symptom‐based diagnoses predominated, with R42 (dizziness and giddiness) and R55 (syncope and collapse) assigned in 20.5% and 7.9% of unweighted visits, respectively. Specific vestibular diagnoses were rare, with H81.1 (benign paroxysmal positional vertigo) and H81.3 (other peripheral vertigo) assigned in 1.4% and < 0.1% of unweighted visits, respectively.

In the adjusted models, predicted probabilities of CT receipt increased with age category: 13.8% (95% CI: 7.2%–20.4%) for < 15 years, 12.9% (95% CI: 8.2%–17.5%) for 15–24 years, 19.1% (95% CI: 15.6%–22.5%) for 25–44 years, 31.9% (95% CI: 27.7%–36.3%) for 45–64 years, 38.1% (33.2%–43.0%) for 65–74 years, and 41.0% (95% CI: 35.7%–46.3%) for ≥ 75 years. Predicted probabilities of antihistamine administration or prescription were 7.2% (95% CI: 2.6%–11.8%) for < 15 years, 6.9% (95% CI: 3.9%–10.0%) for 15–24 years, 11.1% (95% CI: 7.9%–14.3%) for 25–44 years, 6.6% (95% CI: 4.6%–8.5%) for 45–64 years, 4.2% (2.0%–6.4%) for 65–74 years, and 4.1% (95% CI: 2.0%–6.1%) for ≥ 75 years. Predicted probabilities of benzodiazepine administration or prescription were 1.4% (95% CI: 0%–3.2%) for < 15 years, 4.8% (95% CI: 1.4%–8.2%) for 15–24 years, 6.4% (95% CI: 4.6%–8.2%) for 25–44 years, 7.9% (95% CI: 5.9%–9.9%) for 45–64 years, 5.2% (3.1%–7.3%) for 65–74 years, and 4.7% (95% CI: 2.7%–6.6%) for ≥ 75 years.

Our findings regarding CT utilization are notable because neuroimaging for the chief complaint of dizziness rarely results in an actionable neurologic diagnosis and frequently contributes to increased length of stay [7, 8]. While an earlier NHAMCS study reported an 18% CT utilization rate for dizziness from 1993 to 2005 [3], we found a higher CT utilization rate of 28% from 2016 to 2022. Our findings are comparable to another retrospective study finding a 25% CT utilization rate among commercially insured ED dizziness visits from 2006 to 2015 [6], although we note that both rates seem uncharacteristically low in comparison to prospective studies of ED dizziness in which CT rates exceeded 60% [9]. We hypothesize that the low rate of CT utilization in NHAMCS is due to the heterogeneity of the chief complaint of “dizziness,” which contributes to an inflated denominator of total ED dizziness visits because it includes both balance‐related (i.e., vertigo, room‐spinning) and non‐balance related (i.e., lightheadedness, pre‐syncope) symptoms. While clinicians may be able to isolate obvious non‐balance related symptoms of “dizziness” for which neuroimaging is not applicable, NHAMCS relies on pre‐defined variables in the electronic record that cannot capture the nuance required to more precisely isolate balance‐related “dizziness” symptoms.

Still, the relative increase in CT utilization within NHAMCS from 2005 to 2022 is notable [3]. Reasons for this increase are likely multifactorial but may include both patient and clinician factors, such as the desire to rule out a serious neurologic diagnosis. Although evidence‐based physical exam techniques can exclude pathologic diagnoses (e.g., positive Dix‐Hallpike, reassuring HINTS) [10], clinician uptake has been low and inconsistent. Future research should focus on understanding barriers and testing implementation strategies to increase uptake of these evidence‐based exam techniques in clinical practice.

Multiple medications used for symptomatic dizziness, such as meclizine and diazepam, are administered during the ED visit and subsequently prescribed. These medications have been associated with falls and adverse outcomes due to their anticholinergic or sedative properties [4], although we found that antihistamines are used in 7% of ED visits, although this may be an underestimate of actual patient use as antihistamines are available over‐the‐counter, while benzodiazepines are used in 6% of visits. NHAMCS does not report non‐pharmacological interventions delivered in the ED, such as canalith repositioning maneuvers or evaluation by a physical therapist, which are increasingly available in EDs.

Notably, we found that very few ED dizziness visits were given a primary ICD‐10 diagnosis for a specific vestibular condition, such as benign paroxysmal vertigo or vestibular neuronitis; the most common diagnosis was the general symptom‐based code for dizziness and giddiness. The relatively low proportion of BPPV diagnoses in this study (1.4%) is particularly striking compared to the 37.1% BPPV rate from a recent prospective trial of a protocolized diagnostic algorithm for ED dizziness [9] or the 26% BPPV rate reported from a national patient registry [11]. The lack of specific vestibular diagnoses in this study may indicate emergency clinician reluctance or inability to further differentiate specific dizziness etiologies, the inherent non‐specific nature of the patient complaint of “dizziness,” or specific billing and coding practices. The predominance of symptom‐level coding may reflect an opportunity for ED clinicians to improve diagnostic specificity.

A limitation of our study is its retrospective design, which incorporates survey data using pre‐defined variables abstracted from the medical record by reviewers at each participating hospital; these pre‐defined NHAMCS variables tend to include easily obtainable and reliable information (e.g., patient age) but do not capture more complex nuances of ED practice that may drive care patterns, such as patient expectations or referral to the ED from another provider. Similarly, our multivariable models adjusted for a limited number of clinical and demographic covariates present in NHAMCS; there are likely additional important covariates outside of NHAMCS. Lastly, this study contained data only through 2022, which precedes the 2023 publication of the GRACE‐3 clinical practice guideline on acute dizziness and vertigo in the ED [10]. CT utilization rates following publication of the GRACE‐3 may differ given that these guidelines recommend the use of evidence‐based physical exam maneuvers over neuro‐imaging.

In summary, this update of usual emergency care for dizziness spans the contemporary clinical era and covers the final year of NHAMCS data collection. From 2016 to 2022, approximately 1 in 30 U.S. ED visits were for dizziness, with 28% of visits receiving a CT.

## Funding

This work was supported by the National Institute on Deafness and Other Communication Disorders, R21DC022877; National Center for Complementary and Integrative Health, R01AT012367.

## Disclosure

H.S.K. receives an annual stipend from the American Medical Association as Deputy Editor for JAMA Network Open.

## Supporting information


**Data S1:** acem70214‐sup‐0001‐Supinfo1.pdf.

## Data Availability

The data that support the findings of this study are publicly available from the National Center for Health Statistics at: https://www.cdc.gov/nchs/nhamcs/about/index.html.
