# Supplementary material for: U.S. Emergency Department Visits for Dizziness and Vertigo From 2016 to 2022
Source: Acad Emerg Med. 2026 Jan 13;33(1):e70214. doi: 10.1111/acem.70214 (PMC12797808; doi:10.1111/acem.70214)
Supplement: Supplementary file 1 — Data S1: acem70214‐sup‐0001‐Supinfo1.pdf. [file ACEM-33-0-s001.pdf]

eMethods: Usual Care for Dizziness at U.S. Emergency Departments, 2016-2022

NHAMCS Overview: NHAMCS was an annual, national probability sample of U.S. ED visits conducted by the National Center for Health Statistics (NCHS) up to 2022. NHAMCS statistics are derived by a multistage estimation procedure, with sampling weights (cluster, strata, and probability) adjusted for the probability of sample selection and survey nonresponse within time of year, geographic region, and urban/rural and ownership designations, yielding a national probability estimate of ED visit occurrences, percentages, and characteristics. The NHAMCS dataset is publicly available and de-identified and is considered exempt from IRB review.

NHAMCS Data Collection: ED staff at approximately 500 participating hospitals complete a standardized survey instrument for a systematic random sample of patient visits during a randomly assigned four-week reporting period. Data are obtained on demographic characteristics of patients, expected source(s) of payment, patients' complaints, diagnoses, diagnostic/screening services, procedures, medication therapy, disposition, types of providers seen, causes of injury, and certain characteristics of the facility, such as geographic region and metropolitan status.

Medications: From 2014 forward, NHAMCS collected information on up to 30 administered medications and 30 prescribed medications during an ED visit. NHAMCS utilizes the Multum Classification of Therapeutic Classes to record which medications were given for each ED visit. The Multum Lexicon assigns an overall classification based on all ingredients in the medication rather than generic drug codes for each ingredient. Therefore, some medications can have multiple classifications (i.e., diphenhydramine can be classified as both an antihistamine and an antiemetic). The Multum Lexicon assigns a maximum of 4 categories (CAT1-4) to each medication (RX). A given Multum code of interest (e,g., diphenhydramine [d00212]) could have multiple therapeutic/pharmacologic categories (e.g., 070 miscellaneous anxiolytics, sedatives, and hypnotics; 123 antihistamine; 197 anticholinergic antiemetic; 205 anticholinergic antiparkinson agents), each of which could be located in any of the four category positions. Thus, in our code, CAT1-4 were analyzed when determining if that particular medication was administered or prescribed.

Additionally, the Multum Lexicon utilizes a 3-level nested naming system that provides a classification of increasing specificity for each medication (e.g., ondansetron is a Level 1 central nervous system agent, a Level 2 antiemetic/antivertigo agent, and a Level 3 5HT3 receptor antagonist). The most detailed therapeutic level (Multum Level 3) is recorded as a medication’s category (e.g., if RX1 was ondansentron, RX1CAT1 would be coded as a 5HT3 receptor antagonist, not an antiemetic/antivertigo agent). We focused on the following medication classes at Multum Level 3:

Benzodiazepines: 203 benzodiazepine anticonvulsants, 069 benzodiazepines
Antiemetics: 195 5HT3 receptor antagonists, 196 phenothiazine antiemetics, 197 anticholinergic antiemetics, 198 miscellaneous antiemetics, 481 NK1 receptor antagonists,
Antihistamines: 123 antihistamine
Glucocorticoids: 301

We validated our Stata code by exploring antihistamine administration and prescribing in the unrelated ED reason for visit of “allergic reaction.” We selected antihistamines for validation because, based on our clinical experience, are the most commonly utilized medication class for allergic reactions, and because anthistamines can fall under 4 therapeutic categories (as explained above).

Reason for Visit Codes: From 2014 forward, NHAMCS collected information on up to 5 Reason For Visit (RFV) codes. The RFV module-based coding system was created specifically for NHAMCS in 1979; the coding modules can be found in each annual documentation file. We included the NHAMCS RFV code 1225.0 Vertigo – dizziness, which per the NHAMCs codebook includes: “falling sensation, giddiness (dizziness), lightheadedness, loss of sense of equilibrium or balance, room spinning.”

Diagnostic Imaging: NHAMCS has unique indicator variables for the receipt of any computed tomography (CT) scan, or magnetic resonance imaging MRI scan. For MRI, NHAMCS does not have additional indicator variables for MRI type that allow for further specification. For CT, NHAMCS has indicator variables for CT contrast (intravenous contrast), CT abdomen/pelvis, CT chest, CT head, CT other, and CT site not specified. We focused on ED visits for which the CT head indicator variable was positive.
